# Tumour Recurrence, Depth of Invasion, and Temple Location as Independent Prognostic Parameters of Lymph Node Metastases of Head and Neck Cutaneous Squamous Cell Carcinomas

**DOI:** 10.1155/2024/9960948

**Published:** 2024-03-08

**Authors:** Zuzana Horakova, Ivo Starek, Jana Zapletalova, Richard Salzman

**Affiliations:** ^1^Department of Otorhinolaryngology and Head and Neck Surgery, Faculty of Medicine and Dentistry, Palacky University Olomouc and University Hospital Olomouc, I. P. Pavlova 185/6, Olomouc 779 00, Czech Republic; ^2^Department of Medical Biophysics, Faculty of Medicine and Dentistry, Palacky University Olomouc, Hnevotinska 3, Olomouc 775 15, Czech Republic

## Abstract

The excellent survival rate of cutaneous squamous cell carcinoma (cSCC) exceeding 90% is reduced by the presence of nodal metastases by over 50%. We analysed various risk parameters of cSCC to predict the incidence of nodal metastases. A total of 118 patients with the head cSCC were included in a single-institution retrospective study covering the period from 2008 to 2020. Tumour recurrence, temple location, and tumour infiltration depth were found to be independent predictors of nodal metastases (increasing the probability of metastases by 8.0, 8.1, and 4.3 times, respectively). Furthermore, univariate analysis shows that the tumour size and T stage are significant factors increasing the risk of metastases. Several independent risk factors for the development of metastases in the head cSCC have been confirmed. These findings might help identify at-risk patients who require additional attention for adequate radical treatment and close follow-up. In contrast, elective treatment of lymph nodes is not recommended due to the low incidence of regional metastases.

## 1. Introduction

Basal cell carcinomas primarily represent nonmelanoma skin cancers and only one third of this oncological group comprises squamous cell carcinomas (SCC) [1, 2]. Their incidence has increased significantly in the last three decades [3–5]. The reason for this is the increasing age of the population, higher UV radiation exposure due to ozone depletion [4, 5], and a lifestyle with more outdoor activities.

The prognosis of cutaneous squamous cell carcinomas (cSCCs) after adequate surgical treatment is excellent. The 5-year overall survival (OS) exceeds 90% [6, 7]. However, patients with nodal metastases have a poor course, which, as in other head and neck cancers, reduces survival by more than 50%. The five-year OS is only 25–40% [8–13]. The global incidence of nodal metastases in cSCC does not exceed 5% [14, 15]. However, the incidence of cSCC in head and neck regions is higher, commonly reported between 6% and 10% [10, 12, 16] and exceptionally even higher [11]. The reported subclinical nodal involvement usually does not exceed 10% [17–20] and does not reach the 20% limit, which would require prophylactic removal of the associated lymph nodes. The cautious view on these procedures is underlined by the fact that most head cSCC originates from skin regions with lymph drainage to the parotid nodes [5, 15, 21–23]. In addition, total parotidectomy is associated with severe risks (facial nerve and more significant auricular nerve dysfunction, Frey's syndrome, first bite syndrome, and more). Therefore, the National Comprehensive Cancer Network (NCCN) guidelines [24] do not recommend elective neck dissection or parotidectomy.

**Figure 1:**
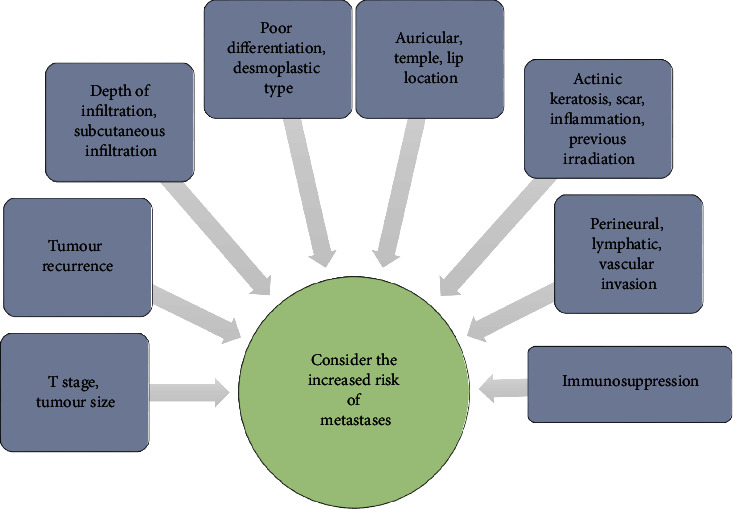
Flow diagram of tumour and patient risk parameters increasing the risk of metastases.

Several lists of potential risk factors for lymphogenic spread were published in relevant literature to stratify the risk of metastases incidence [9, 24–28] (Figure 1).

Identifying predictors of a higher risk of local recurrence and metastases has implications for tumour staging.

Individual authors have identified several tumour and patient characteristics that increase the risk of metastatic incidence. Since 2009, the TNM classification system has been adjusted to account for the presence of the following two or more additional risk factors: >4 mm depth, Clark level V, perineural invasion, angioinvasion, localisation on the ear or lip, and poorly differentiated or undifferentiated tumours. The presence of these risk factors upstages the T value [29, 30].

However, the results of various independent studies have not always been consistent, and the predictive values of the factors tested and their combinations have not been strong enough to serve as independent prognostic markers.

In our retrospective study, we analysed a group of 118 patients with cSCC of the face, auricular, and frontotemporal regions to identify predictors of nodal metastases and patient prognosis.

## 2. Patients and Methods

### 2.1. Patient and Methods Database

A total of 118 patients with cSCC were included in the retrospective study covering the period from 2008 to 2020. Of these patients, 78 were already treated with the primary cSCC at our clinic, and the remaining 40 were referred from other institutions due to disease recurrence involving regional lymph nodes (22 cases), the site of the primary tumour (5 cases), or both (13 cases).

The clinical stage of the disease was determined in all patients before initiating their treatment. The skin tumour was evaluated by a dermatologist, and cervical and parotid nodes were examined clinically using imaging methods (US, CT, MRI, and PET/CT). The histopathological diagnosis of squamous cell carcinoma was verified postoperatively by examining surgical specimens or biopsies of inoperable tumours. The lymph node status was assessed in clinically evident nodal metastases using aspiration cytology (only in cases where the diagnosis was later confirmed through histology after surgical treatment) or core needle biopsy.

In our study, we included only those risk factors listed in the NCCN recommendation [24] for which we had a sufficient amount of relevant data necessary for valid statistical analysis, i.e., the clinical stage of the tumour, tumour size (measured in millimetres at the greatest diameter) and depth of its invasion (measured in millimetres) or invasion into the subcutaneous layer, grade of differentiation (G1- G3), perineural invasion, and resection margins (R). In Rx and R1 cases, the completeness of resection, i.e., R, was assessed through a histological review of the surgical specimen harvested during revision procedures performed immediately after the primary operation. The predictive power of the R parameter was evaluated only in those 35 cases where metastases appeared as a relapse. R0 tumours were categorised based on resection margins using a 4-mm cutoff. Other factors (angioinvasion, perineural spread, and similar) were not analysed.

**Figure 2:**
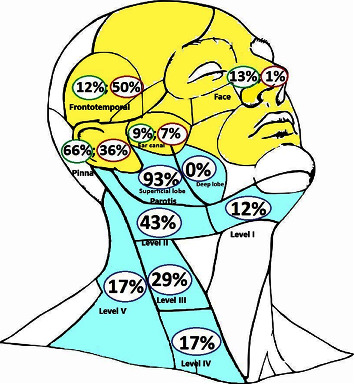
Distribution and frequency of primary skin carcinomas and their regional metastases.

Within the entire set of 118 tumours, 76 were evaluated as cN0. This group consisted of 69 men and 7 women, aged 41–92 years (average of 74 years). The tumours were located on the skin of the auricle in 50 patients, on the external auditory canal in 7 patients, frontotemporally in nine patients and on the nose in 10 patients (Figure 2). The follow-up period ranged from 3 to 192 months (average of 48 months).

Metastatic nodal involvement was found in 42 patients (34 men and 8 women) aged 41–95 years (average of 79 years). Only three cases showed involvement of cervical nodes (the areas I–III), 24 cases involved only the parotid gland, and 15 cases involved both localities (5 times in level II, 3 times in levels II and III, once in levels II and V, and 6 times in 4 and more neck levels) (Figure 2).

The stage of regional nodal metastases was as follows: cN1 in two patients, cN2 in five patients, and cN3b, all with the extracapsular extension, in 35 patients.

**Figure 3:**
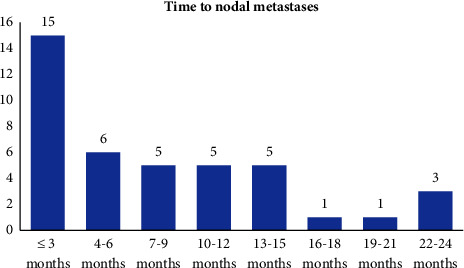
Time between skin tumour excision and nodal metastases manifestation.

In 35 patients, nodal metastases occurred within the range of 3–24 months (average of 8 months), and 10 of these cases also presented with local tumour recurrence (Figure 3). In 7 cases, nodal metastases were already present during the clinical presentation of the primary tumour. The patients were followed up for 2–125 months (an average of 31 months). The primary tumour was located frontotemporally in 21 patients, on the auricle in 15 patients, in the external auditory canal in 3 patients, and on the external nose, neck, and lower lip in 1 patient each (Figure 2).

Clinical and demographic data of all the patients, including the tumour stage, tumour size and depth of invasion, histopathological grading, quality of resection margins, perineural invasion, other skin pathology (keratic acanthosis), and immunosuppression are presented in Table 1 (Table 1).

### 2.2. Statistical Evaluation

This study compared 5-year overall and disease-free survival in subgroups of patients with and without nodal metastases. To predict nodal metastases of primary tumours, we assessed the importance of the following five parameters: T stage according to the current WHO TNM classification [27], tumour size, depth of skin invasion (greater than or equal to 4 mm), histological differentiation (G1/2 vs. G3/4), microscopic resection margins (negative), and local recurrences (more than three months after remission). Negative resection margins were determined based on the absence of microscopic evidence of malignant cells, with close resection margins defined as equal to or smaller than 4 mm.

Fisher's exact test was used to compare the incidence of risk factors in patients with metastases and those with nonmetastatic tumours. The study also utilised Kaplan–Meier analysis with the log-rank test to compare OS and disease-free interval (DFI) between the two groups and to evaluate the impact of risk factors on OS and DFI.

The significance of individual parameters for lymphogenic spread was evaluated using univariate analysis. An algorithm was defined using Cox stepwise logistic regression analysis to determine the predictive ability of significant parameters to calculate the risk of nodal metastases in individual tumours. Due to the patient group size (*n* = 118), only the three most significant parameters from the univariate analysis were evaluated.

IBM SPSS Statistics version 23 (Armonk, NY: IBM Corp.) was used for data analysis. All tests were conducted at a significance level of 0.05.

## 3. Results

### 3.1. Treatment of cN0 Patients

In two patients, the primary tumour was treated with radiotherapy. Furthermore, 76 patients underwent surgery, with 51 achieving adequate resection margins and 25 having close or positive margins. Five patients with positive or close margins underwent reresection (three patients) or radiotherapy (two patients). Local relapse occurred in six patients, and dissemination occurred in one patient. No nodal recurrence was recorded.

### 3.2. Treatment of cN + Patients

Among 42 patients with nodal metastases, the primary tumour was treated with radiotherapy in six cases. Surgical resection was performed on 36 patients, 21 with insufficient resection margins. Consequently, 20 patients underwent reoperation, and two received adjuvant radiotherapy. The remaining patient underwent radiotherapy. Of the three patients with solely cervical metastases, one underwent comprehensive neck dissection, another patient underwent selective (II-IV) neck dissection, and the remaining patient received radiotherapy. On 17 out of 24 patients with isolated parotid metastases, near-total or total parotidectomy was performed along with elective neck dissection of levels II–IV. Microscopic examination of all patients' specimens revealed no evidence of subsequent metastases. The remaining seven patients received only palliative radiotherapy. On 9 out of 15 patients with concurrent parotid and cervical metastases, parotidectomy and curative neck dissection were performed (3x comprehensive and 6x selective). Adjuvant radiotherapy was performed on 12 patients. Primary radiotherapy (5x) or symptomatic treatment (1x) was indicated due to poor general condition in six patients.

Remission was achieved in 23 out of 26 patients who received curative treatment for metastatic disease (i.e., 9x surgery, 17x surgery with adjuvant radiotherapy (RT), or chemoradiotherapy (ChRT)). Eight patients experienced relapse (six in the parotid, one in the cervical nodes, and one with distant metastasis).

### 3.3. Treatment Results

In the group of 74 surgically treated patients with N0 cSCC, i.e., without clinically evident (63 patients) or histologically confirmed (11 patients) lymph node metastases, the probability of a 3-year recurrence-free interval was 98%. Overall survival was 97%, with a median survival or relapse time of 159 months (CI 95%: 134–184) and 150 months (CI 95%: 126–175), respectively.

**Figure 4:**
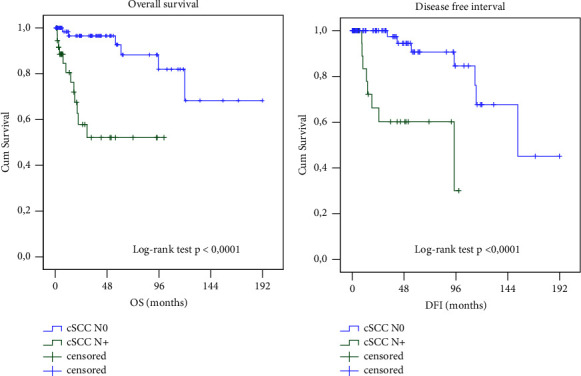
Overall survival prediction (a) and disease-free interval prediction (b) (Kaplan–Meier) in groups of patients with and without nodal metastases (cSCC N+ and cSCC N0).

In the group of 42 patients with metastatic cSCC, the probability of 3-year overall survival was 52% and the recurrence-free interval was 60%. The average survival time and time to recurrence were 59 months (CI 95%: 42–77) and 64 months (CI 95%: 45–83), respectively. These differences in both parameters were statistically significant (*p* < 0.0001). Cox regression analysis confirmed a much higher risk of death (8.1-fold, 95% CI: 2.8–23.6) and recurrence (8.8-fold, 95% CI: 2.6–29.6) in metastatic tumours (Figure 4).

No difference was observed when comparing early stages (N1 + 2) to advanced stages (e.g., N3b) with the extracapsular spread (*p*=0.727). However, there was an obvious imbalance in patient distribution between the two groups, with the most advanced stage of nodal metastases with the extracapsular spread being predominant (35 patients). In contrast, early stages (N1-2) were diagnosed in only seven patients.

In comparison to nonmetastatic carcinomas, metastatic carcinomas showed a significantly higher incidence of recurrences (*p*=0.00005), tumour infiltration depth greater than 4 mm (*p*=0.00018), tumour invasion to the subcutaneous layer (fat or cartilage) (*p*=0.002), higher stages classified by T value (above T1) (*p*=0.00013), larger tumours (size >10 mm (*p* < 0.0001), tumour located on the skin of temporal region (*p* < 0.0001), perineural invasion (*p*=0.021), as well as microscopically positive and close margins (*p*=0.014), and a significantly lower incidence of tumours located on auricle (*p*=0.002).

No significant difference was found in terms of histopathological grading of cSCC in both groups (*p*=0.118), presence of actinic keratosis (*p*=0.564), or incidence of multiple previous skin malignant tumours (both basaliomas and spinaliomas) in other locations of the head and neck (*p*=0.061), although the latter parameter was nearly significant (Table 2).

### 3.4. Predictors of Nodal Metastases

Univariate logistic regression analysis revealed several significant predictors of lymph node metastases. These included T stage (*p*=0.001), tumour size over 10 mm (*p* < 0.0001), tumour location (*p*=0.0002), temporal tumour location precisely (*p* < 0.0001, OR: 7.78), tumour recurrence (*p*=0.00025, OR: 7.89, and CI: 2.6–23.8), tumour invasion depth (≥4 mm) (*p*=0.00029, OR: 4.54, and 95% CI: 2.0–10.3), subcutaneous invasion of the tumour (*p*=0.00029, OR: 4.54, and CI: 1.62–10.6), close and positive resection margins (*p*=0.026, OR: 2.62), and perineural invasion (*p*=0.038, OR: 10.13, and CI: 1.14–89.9). The statistical significance of the T stage and tumour size was the most substantial (*p*=0.0001 and *p*=0.003) for auricular tumours. The importance of histological grading of cancer was suggestive but it did not reach a significant level (*p*=0.121, OR: 1.93, and CI: 0.84–4.42) (Table 3).

### 3.5. Independent Predictors of Lymph Node Recurrence

Stepwise logistic regression analysis evaluated the three most significant parameters and confirmed that tumour recurrence, tumour infiltration depth, and temporal tumour location were independent predictors of nodal metastases recurrence. Temporal skin tumour location increased the likelihood of metastases by 8.1 times (95% CI: 2.86–22.9), tumour recurrence by 8 times (95% CI: 2.3–27.6), and depth of tumour invasion (>4 mm) by 4.3 times (95% CI: 1.64–11.2) (Nagelkerke R square = 0.570) (Table 4).

The negative predictive value (NPV) and the positive predictive value (PPV) of the significant risk parameters for the prediction of metastases are summarised in Table 5.

### 3.6. Early Tumours

The predictive significance of the parameters mentioned above was independently verified in early (T1-2) tumours, showing a significantly lower incidence of lymph node metastases compared to T3-4 tumours (*p*=0.001). Within the T1-2 subgroup, N+ tumours showed a significantly higher incidence of recurrence (*p*=0.003), more significant invasion (*p*=0.0004), and higher grade of differentiation (*p*=0.013) compared to N0 tumours. The results of univariate logistic regression confirmed the significance of recurrence (*p*=0.003, 95% CI: 1.9–28.2), depth of tumour invasion (*p*=0.001, 95% CI 2.1–16.8) and, in contrast to the results in the entire study group, carcinoma differentiation (*p*=0.016, 95% CI: 1.3–9.8) for the development of lymph node metastases. Stepwise logistic regression analysis further verified the independence of the first two predictive parameters. Recurrence increased the risk of nodal metastases by 6.98 times (95% CI: 1.65–29.6) with NPV of 78% (95% CI: 73–83) and PPV of 67% (95% CI: 40–86). Depth of tumour invasion increased the risk by 5.8 times (95% CI: 1.9–17.4) with NPV of 86% (95% CI: 77–92) and PPV of 49% (95% CI: 37–60).

## 4. Discussion

### 4.1. Aetiology and Diagnostics of cSCC

Cutaneous SCC arises from actinic keratosis (AK) through an uncontrolled proliferation in a long-term, multistep process. Typical histological features of SCC include parakeratosis, hyperkeratosis, nuclear pleomorphism, atypical mitoses, and multinucleated cells through all epidermal layers [49, 50].

The diagnosis of cSCC must always be established histologically through biopsy or excision. Depending on the tumour size, biopsy may be performed before radical extensive surgical treatment of a large tumour.

Histological examination with H&E staining is used to confirm the diagnosis; immunohistochemical testing is added only in rare cases of uncertain diagnosis. Various histological variants of cSCC with different prognoses and metastatic incidences can be distinguished. Desmoplastic, acantholytic, and adenosquamous variants of SCC are known to have worse prognosis with highly infiltrative growth, perineural and perivascular spread, and a high incidence of metastases [42, 51, 52].

However, as these types were rarely identified in our set of metastatic tumours, their significance was not analysed.

SCC is usually diagnosed based on its typical appearance; dermoscopy can be helpful for small lesions that may be confused with keratoacanthoma. Bowen disease is another specific entity, an intraepidermal carcinoma with atypia of keratinocytes at all levels of the epidermis (carcinoma in situ). The early tumour stage can be missed if the tumour develops within a chronically inflamed area accompanied by pseudoepitheliomatous hyperplasia in the surrounding tissue [53].

In our study, the histology of the skin tumour was confirmed in all cases by examining the resection specimen. A pretreatment diagnostic biopsy was performed in only a few advanced cases. Parotid and neck nodal metastases were always diagnosed by standard HE histological examination, which was performed either on tissue samples retrieved after open biopsy or through preoperatively obtained core cuts, and the diagnosis was subsequently confirmed postoperatively.

### 4.2. Incidence, Location, and Prognostic Impact of Regional Metastases

The reported incidence of lymph node metastases in cSCC across all tumour sites is generally very low. In recent population studies, Veneblas [47] reported a 3-year cumulative incidence of metastases of 2% in a cohort of 76,977 patients, and Tokezs reported a 10-year cumulative incidence of only 1.9% in a study of 11,137 patients [48]. However, in head and neck cSCC, the risk of metastases is usually higher, up to 6% [8, 11, 16, 26, 35]. The incidence reported in independent studies on a limited cohort of patients can vary widely, ranging from 10% to 21% [8, 9, 11, 35].

In our study, metastases were present in 35.6% (42/118) cases. This high incidence compared to the previously published studies is probably due to selection bias. Our cohort of patients is not population based but reports on the results of a tertiary ENT centre to which most patients with cN+, advanced tumours, or otherwise complicated patients are referred.

Similarly to other authors [8–13, 26–28], we demonstrated a significant impact of these metastases on survival and recurrence-free interval. Their presence increased the risk of death and recurrence 8.1-fold and 8.8-fold, respectively.

### 4.3. Lymphatic Spread

When assessing the anatomical localisation of lymph node metastases in cSCC, it is important to note that the pattern of lymph drainage can vary. As described by several authors, for most head skin locations (e.g., auricular, temporal, zygomatic, frontal, and anterior scalp), the first lymph node basin is the parotid and periparotid regions.

The involvement of the neck lymph nodes typically follows the parotid region. In addition, the external jugular nodes can be involved in either the parotid or II neck level, which is significant [12, 21–23, 36].

Our results supported the findings of these authors. In our set of cases, 39 out of 42 showed metastases to the parotid gland, whereas only three patients had metastases restricted to the neck lymph nodes, with clinically negative involvement of the parotid gland. The primary skin tumours in these cases were located on the skin of the mental region, the apex of the external nose, and the skin of the lateral neck.

However, in 24 out of 39 cases, the metastases were confined to the parotid nodes alone, whereas parotid and neck lymph node metastases were present in only 15 cases. Regarding the neck levels, the metastases were most frequently located in the upper jugular nodes (level II), followed by the middle jugular nodes (level III). (Figure 1).

The extracapsular extension (ECE) of nodal metastases is generally considered a negative prognostic predictor, which has been confirmed in several clinical studies using univariate analysis [23, 37].

Our results did not confirm this assumption, as the univariate analysis did not reveal a significant association between ECE and worse prognosis. An uneven distribution between the groups may have influenced the statistical results, as ECE was confirmed in most cases (35 out of 42).

### 4.4. Tumour Localisation

In general, SCCs located on the skin of the head and neck area are more likely to metastasise compared to those on the trunk and extremities. This risk also varies depending on the specific skin locations of the head, of which the ear and periauricular, temporal regions, lips, and vermilion have the highest risk of metastases.

When comparing tumours located in different areas, those on the skin of the temporal area had a higher risk of metastases in our cohort. Furthermore, according to regression analysis, the localisation of tumours in the temporal area was confirmed as an independent prognostic factor that increases the risk of metastases 8-fold; these findings are consistent with independent studies by Haisma and Brougham [8, 16] as well as a meta-analysis by Thompson [10].

However, our results did not confirm the previously observed high risk of metastases of tumours localised on the skin of the ear. We believe that this discrepancy is due to a tumour size bias. The frequency of small T1 tumours was significantly higher on the skin of the auricle than in any other location (*p*=0.0001) [8, 10, 16, 26, 31, 35, 44]

### 4.5. Tumour Stage

Our study confirmed the significant importance of the T stage, specifically the tumour size, as an independent predictor of nodal metastases [9–12, 26, 28, 30–34, 44].

According to our results, any stage higher than T1 is considered significant. The association between the T stage and nodal metastases was demonstrated in previous meta-analyses by Rowe [26] and, more recently, by Zeng [28], who evaluated 29,000 skin cSCC of the head and neck from 43 studies. A meta-analysis by Thompson [10] also obtained similar results in skin cancers of the head and neck regions.

In our study, the risk of developing lymph node metastases in T2 and T3/T4 carcinomas was significantly increased compared with T1 tumours.

### 4.6. Tumour Size

Tumour size, as one of the parameters included in the TNM staging system, is considered a risk factor related to the occurrence of lymph node metastases. Several studies have already confirmed this assumption [8, 26, 32, 35]. The critical tumour size estimated by several authors [8, 11, 26] and also accepted by the NCCN panel is 20 mm for all skin sites [24].

In head and neck cSCCs, even smaller tumours are considered high risk; the critical diameter was assumed to be 15 mm by Quaedvlieg and 6 mm by Bratsch [32, 35]. Our study confirmed that any tumour exceeding 10 mm in its greatest diameter was associated with a significantly higher risk of developing nodal metastases.

### 4.7. Tumour Recurrence

Recurrence is strongly associated with the risk of metastases. Cox regression as an independent predictor confirmed this relationship's strength, which increased the risk of metastases by 7.6 times. Numerous authors have extensively documented similar results supporting a strong association between tumour recurrence and the development of metastases [9, 11, 34].

### 4.8. Depth of Tumour Invasion

Our study demonstrated that a depth of tumour invasion above 4 mm and local recurrence are independent factors associated with nodal metastases, increasing the risk by 4.3 and 7.4 times, respectively. These findings correspond to previously published studies [8–11, 16, 26, 27, 31, 35].

In their meta-analyses, Thompson [10], Haisma [8], and Cherpelis [11] stated that the risk of regional lymph node involvement increases with the depth of invasion, starting from 2 mm and beyond. This finding is consistent with the results of Brantsch's analysis [35], which indicates that tumours (regardless of the T stage) with an invasion of 2 mm do not establish nodal metastases, while tumours infiltrating to a depth of 2–6 mm and above 6 mm metastasise in 4% and 16%, respectively. Our study confirms that the depth of invasion is a crucial factor in nodal metastases, even in early-stage (T1 and T2) cancers.

The depth of tumour invasion was also classified by Clark by the infiltrated layer of the dermis or subdermis. The risk of metastases increases substantially with the invasion of cartilage, subcutaneous fat, or bony structures.

Univariate analysis confirmed the expected relationship at a significant level (*p*=0.003) although the factor was not estimated as an independent predictor. Numerous previous studies have also shown a positive correlation between the involvement of subcutaneous layers and the risk of metastases [9, 11, 12, 30, 32, 34].

### 4.9. Histological Differentiation

A low degree of histopathological differentiation was found to be significantly associated with metastases in T1 and T2 tumours (*p*=0.016) in the univariate analysis but not in the multivariate analysis. In more advanced cancers (T3/T4), there was only an indication of a higher risk of lymph node spread. However, the results of published studies on this subject are controversial. While Clark [12], similar to our findings, did not confirm the association between the grade of differentiation and the occurrence of nodal metastases, other authors have demonstrated it at statistically significant levels [8, 9, 16, 22, 26, 30–32, 34, 44].

These differences might be explained by the grading heterogeneity in large volumes of advanced tumours, which can contain areas with varying degrees of differentiation. Standards for determining grading in these carcinomas need to be provided.

In general, poor differentiation (G3) significantly increases the risk of metastases. However, an association for moderate grade (G2) has also been reported [8].

### 4.10. Actinic Keratosis, Prior Radiotherapy Site, Chronic Inflammation

AK is a nontumorous pathology that can progress into invasive cutaneous squamous cell carcinoma. The progression of AK to SCC is unpredictable, and SCC may develop even without specific signs of lesion progression [43].

AK, as well as chronic scarring, previously irradiated terrain, or inflammation, increases the risk of metastases of cSCC that developed in these conditions.

We confirmed this association by observing a higher frequency of these pathologies in patients with metastatic tumours. The frequency of patients with multiple previous cSCCs was almost significantly higher in the cN + SCC group [11, 26, 32, 43, 54, 55].

### 4.11. Perineural Invasion and Lymphatic or Vascular Involvement

Perineural invasion is another biological factor that increases the risk of metastases development [10, 11, 16, 26, 30, 44, 45].

There is a particularly high risk associated with the involvement of a nerve located deeper than the dermis or measuring ≥0.1 mm [46, 56].

If perineural invasion (PNI) is suspected, an MRI should be performed [56].

Our results confirmed this observation, showing a significant association between PNI and the incidence of metastases (*p*=0.038) in the univariate analysis. However, due to the low frequency of PNI (12% in cN vs. 1.3% in cN0, *p*=0.021), this factor was not included in the regression analysis.

The involvement of lymphatic or blood vessels is another parameter in skin tumours that plays a role in the development of metastases [44, 46, 56]. However, this factor was diagnosed in exceptional cases among our patients with metastases and could not be analysed.

### 4.12. Immunosuppression

From the patient's perspective, immunosuppression worsens the prognosis and increases the incidence of metastases. Nowadays, immunosuppression, mostly of iatrogenic origin in transplant recipients, is becoming an important factor [10, 26, 30, 47, 48, 57]. Nevertheless, our study included only one immunosuppressed patient in each group (cN0 and cN+), which limits further statistical analysis.

### 4.13. Resection Margin

Univariate analysis showed that the positivity of microscopic resection margins was a significant predictor of nodal metastases in cSCC of the head and neck. Other authors [36, 38] have obtained similar results for tumours in this and other locations. According to Mourouzis, this parameter is an independent predictor [39].

We did not find a relationship between close margins (less than 4 mm wide horizontal resection) and the incidence of nodal metastases. Griffiths [58] and Quaedvlieg [32] have also reached the same conclusions, considering the vertical dimension, i.e., the bed of resection, separately. According to Jenkins [40] and Stratigos [41], tumours with a resection margin of less than 2 mm tend to metastasise to lymph nodes, making this a critical margin.

Distribution and frequency of 76 nonmetastatic (green) and 42 metastatic (red) primary cSCC in the frontotemporal, auricular region, external ear canal and face, and regional nodal metastases (blue) of 42 patients in superficial and deep parotid lobes and neck regions were found (Table 6).

## 5. Conclusion

The tumour recurrence, temple location, depth of invasion, size, and T stage were found to be the most relevant independent predictors of lymph node metastases in cSCC of the head and neck. Elective lymph node dissections might improve the poor prognosis of metastatic cancer patients. However, the predictive values of the clinical and histological parameters assessed are insufficient to justify elective surgery. We suggest that a more accurate examination of molecular markers should be performed to improve predictive accuracy.

## Figures and Tables

**Table 1:** Characteristics of a patient group and tumour risk parameters.

		cSCC N+	cSCC N0
Number of patients		42	76

Sex	Male	34	69
	Female	8	7

Age	Min-max; mean	41–95; 79	41–92; 74

Skin tumour T stage	T1	13	54
	T2	11	8
	T3	14	13
	T4	4	1

N stage	N0		76
	N1	2	
	N2	5	
	N3	35	

Stage	I		54
	II		8
	III	2	13
	IV	40	1

Tumour size (mm)	<10	5	38
	10–20	14	17
	20–40	17	15
	>40	5	6

Grade of SCC differentiation	G1	3	35
	G2	24	24
	G3	15	17

Resection margins	Positive R+	6	3
	Uncertain Rx	5	1
	Close (≤4 mm) R0	7	21
	Save ≥4 mm R0	14	51

Depth of invasion	≥4 mm	30	27

Recurrence		15	5

Tumour site	Auricular	15	50
	External ear canal	3	7
	Temporal	21	9
	Nose	3	10

**Table 2:** Differences between the cSCC N0 and cSCC N+ groups of patients.

		cSCC N+		cSCC N0		Fisher's exact test's *p* value
		Number	Percent (%)	Number	Percent (%)	
Recurrence	0	27	64.3	71	93.4	0.00005
	1	15	35.7	5	6.6	

Depth of invasion ≥4 mm	0	12	28.6	49	64.5	**0.00018**
	1	30	71.4	27	35.5	

Grading G 1 + 2 vs. G3	0	27	64.3	59	77.6	0.11800
	1	15	35.7	17	22.4	

Resection margins R1, Rx, R0	R0	14	43.8	51	67.1	**0.01400**
	R1	6	18.8	3	3.9	
	Rx	12	37.5	22	28.9	

Resection margins R0 vs. R1 + Rx	R0	14	43.8	51	67.1	**0.02400**
	R1 + Rx	18	56.3	25	32.9	

T stage	1	13	31.0	54	71.1	**0.00013**
	2	11	26.2	8	10.5	
	3	14	33.3	13	17.1	
	4	4	9.5	1	1.3	

T stage 1 vs. 2 vs. 3 + 4	1	13	31.0	54	71.1	**0.00014**
	2	11	26.2	8	10.5	
	3 + 4	18	42.9	14	18.4	

T stage 1 + 2 vs. 3 + 4	1 + 2	24	57.1	62	81.6	**0.00400**
	3 + 4	18	42.9	14	18.4	

Tumour size (mm)	<10	5	11.0	38	50.0	0.0002
	10–20	14	33.3	17	22.4	
	20–40	17	40.5	15	19.7	
	>40	5	14.3	6	7.9	

Tumour size (mm)	<10	5	11.9	38	50.0	**<0.0001**
	10–20	14	33.3	17	22.4	
	>20	23	54.8	21	27.6	

Tumour site	Auricular	15	35.6	50	65.8	**<0.0001**
	External ear canal	3	7.1	7	9.2	
	Temporal	21	50.0	9	11.8	
	Nose	3	7.1	10	13.2	

^∗^bold values are statistically significant.

**Table 3:** Univariate logistic regression: nonadjusted OR of cSCC risk factors for nodal metastases.

	*p*	OR	95% CI	
			Lower	Upper
Recurrence	0.00025	7.89	2.61	23.82
Depth of invasion ≥ 4 mm	0.00029	4.54	2.00	10.28
Poor differentiation	0.121	1.93	0.84	4.42
Resection margins R1, Rx, R0	0.025			
Rx vs. R1	0.010	7.29	1.62	32.9
Rx vs. R0	0.143	1.99	0.79	4.98
R0 vs. Rx + 1	0.026	2.62	1.13	6.12
Temporal location	<0.0001	7.44	2.96	18.7
Tumour size (mm) (10-mm referral)	0.002			
<10 vs. 10–20	0.002	6.26	1.94	20.17
<10 vs. 20–40	0.0003	8.61	2.69	27.54
<10 vs. > 40	0.007	7.60	1.75	32.93
Tumour size (mm) (10-mm referral)	0.001			
<10 vs. 10–20	0.002	6.26	1.94	20.17
<10 vs. > 20	0.0002	8.32	2.76	25.11
T stage 1, 2, 3, 4	0.001			
T2 vs. 1	0.002	5.71	1.91	17.1
T3 vs 1	0.002	4.47	1.70	11.8
T4 vs 1	0.015	16.62	1.71	161.4
T stage 1, 2, 3 + 4	0.000			
T2 vs. 1	0.002	5.71	1.91	17.1
T3 + 4 vs. 1	<0.0001	5.34	2.12	13.46
T stage 1 + 2, 3 + 4	0.005	3.32	1.43	7.71

**Table 4:** Stepwise logistic regression of cSCC risk factors for nodal metastases.

	*p*	OR	95% CI	
			Lower	Upper
Recurrence	0.001	8.73	2.38	32.02
Depth of invasion ≥4 mm	0.011	4.08	1.38	12.01
Temporal location	<0.0001	8.09	2.86	22.9

**Table 5:** Positive predictive values (PPVs) and negative predictive values (NPVs) of cSCC risk factors for nodal metastases.

	PPV (%)	95 (%) CI	NPV (%)	95 (%) CI
Recurrence	75	54%–89	72	68%–77
Depth of invasion ≥4 mm	53	44%–61	80	71%–87
Resection margins Rx + 1 vs. R0	42	32%–53	79	71%–85
Temporal location	70	51%–82	76	66%–85
T stage: T 3 + 4 vs. 1	56	43%–69	81	73%–87

**Table 6:** List of cSCC risk factors and their significance for incidence of metastases in our patients and in previously published studies.

Risk parameters	Our results of statistical analysis	Studies confirming a significant risk for incidence of metastases
T stage and tumour size	Significant	Haisma [8], Wermken [9], Thompson [10], Cherpelis [11], Clark [12], Rowe [26], Zeng [28], Jambusaria-Pahlajani [30], Lehnerdt [31], Quaedvlieg [32], Roozeboom [33]

Tumour recurrence	Significant	Wermken [9], Cherpelis [11], Moore [34]

Depth of infiltration	Significant	Haisma [8], Wermken [9], Thompson [10], Cherpelis [11], Brougham [16], Rowe [26], Leibovitch [27], Brantsch [35], Lehnerdt [31], Quaedvlieg [32], Roozeboom [33]

Subcutaneous infiltration	Significant	Wermken [9], Cherpelis [11], Clark [12], Jambusaria-Pahlajani [30], Moore [34], Quaedvlieg [32]

Positive resection margins	Significant	Wermken [9], Lee [36], Ch'ng [37], Quaedvlieg [32], Genders [38], Mourouzis [39],

Close resection margins	Not significant	Jenkins [40], Stratigos [41]

Poor differentiation	Not significant	Haisma [8], Wermken [9], Clark [12], Brougham [16], Vauterin [22], Rowe [26], Zeng [28], Jambusaria-Pahlajani [30], Karia [37], Lehnerdt [31], Moore [34], Quaedvlieg [32]

Desmoplastic type	Not evaluated	Breuninger [42]

Actinic keratosis	Not significant	Cherpelis [11], Rowe [26], Moore [34], Quaedvlieg [32], Del Regno [43]
Inflammation		
Scarring		
Irradiation		

Temple location	Significant	Haisma [8], Thompson [10], Brougham [16]

Ear location	Not significant	Haisma [8], Wermken [9], Thompson [10], Rowe [26], Brantsch [35], Karia [44], Lehnerdt [31],

Perineural invasion	Not evaluated	Thompson [10], Cherpelis [11], Brougham [16], Rowe [26], Jambusaria-Pahlajani [30], Ch'ng [37], Karia [44], Moore [34], Quaedvlieg [32], Lin [45], Carter [46]

Angioinvasion	Not evaluated	Quaedvlieg [32]

Immunosuppression	Not evaluated	Cherpelis [11], Rowe [26], Venables [47], Tokez [48], Ch'ng [37]

Lymphatic invasion	Not evaluated	Moore [34]

## Data Availability

The on-patient individual clinical and pathological details used to support the findings of this study are available from the corresponding author upon request.
